# Disaster-related deaths: Interpretation as an indicator of the medium-to-long-term impact of disaster and its caveats

**DOI:** 10.7189/jogh.14.03030

**Published:** 2024-09-20

**Authors:** Momoka Yamamura, Tianchen Zhao, Chika Yamamoto, Toshiki Abe, Chihiro Matsumoto, Masaharu Tsubokura

**Affiliations:** Department of Radiation Health Management, Fukushima Medical University School of Medicine, Fukushima, Fukushima, Japan

On 1 January 2024, a 7.6-magnitude earthquake accompanied by a tsunami struck the Noto region of Ishikawa Prefecture in the Hokuriku region in Japan. As a result, 4757 buildings in Ishikawa Prefecture were partially or completely destroyed. Furthermore, the death toll reached 240 individuals as of 5 February 2024, with 60% of the fatalities involving older individuals aged ≥70 years. Regarding disaster situations in communities with a significant proportion of older individuals, such as this one, there is considerable social attention and frequent media coverage of the number of disaster-related deaths. As of 5 February 2024, 15 reported indirect deaths have been attributed to the 2024 Noto peninsula earthquake.

Disaster-related deaths can result either from direct or indirect disaster impacts. Direct deaths are those caused by the direct physical effects of disasters, such as earthquakes, tsunamis, and (external and internal) radiation exposure after nuclear accidents. Indirect deaths have been reported to occur due to the following causes: disruption in the health care system at the community level; social isolation, stress, and anxiety; social change and neglect due to social isolation, stress, and loss of financial support; and exacerbation of underlying diseases due to interruption of health care delivery services. These are considered surrogate markers for the indirect and secondary effects of a disaster. For example, in the case of the Great East Japan earthquake and the Fukushima Daiichi nuclear power plant accident in March 2011, 3794 people died from disaster-related causes. Remarkably, in the Fukushima Prefecture, the number of individuals who died indirectly due to the consequences far exceeded the number of those who directly lost their lives [1].

Thus, disaster-related deaths are frequently used to assess the impact of disasters; preventing disaster-related deaths is a crucial aspect of public health responses that require medium-to-long-term strategies. However, when interpreting data on disaster-related deaths in Japan and developing public health measures, careful attention must be paid to several aspects when evaluating these figures.

In Japan, the concept of ‘disaster-related deaths’ was not initially established as a metric for comprehending the impact of disasters but rather created in August 1967 following the Uetsu heavy rainfall to provide disaster relief payment to the victims’ families [2]. Unlike the USA, where physicians record disaster-related deaths on death certificates, which is followed by an investigation by the Federal Emergency Management Agency, Japan’s process involves individual certification committees at the municipal level [3]. These committees determine disaster-related deaths based on various factors including medical, legal, and social considerations. Highlighting that these assessments vary depending on individual municipalities and disasters is crucial.

Second, based on the system by which disaster-related deaths are certified through review applications by bereaved families, the number of reported deaths may underestimate the actual number of individuals who died from disaster-related causes. For example, in the case of an older person living alone who passes away, the death may not be included in the tally of disaster-related deaths if there are no surviving family members. Moreover, even if surviving family members are present, bureaucratic procedures and documentation requirements for a certification application could be substantial, making some individuals hesitate to initiate the application process. As a result, there is concern that the real number of deaths attributed to disaster-related causes may be underestimated.

Third, owing to privacy concerns, Japan has no comprehensive database of disaster-related deaths. Additionally, the location of data and whether or not to disclose this data are determined by each municipality. Although some municipalities disclose detailed information on disaster-related deaths, others do not even release basic information such as sex and age. There is no clear requirement for obtaining the bereaved family's permission before disclosure. However, some administrations mistakenly consider it necessary. As a result, valuable data that has been collected is not being disclosed or utilised to improve future responses to disasters. In the case of the Fukushima Daiichi nuclear accident, our team obtained detailed data from Minamisoma and publicly disclosed the analysis results based on this data [4–6]. According to this research, vulnerable populations who face greater challenges in being independently evacuated compared with the general population and require assistance, such as people with visual and hearing disabilities, those with physical disabilities, infants, older people, injured and hospitalised cases, pregnant women, foreign individuals, and others who have difficulty being evacuated on their own are particularly susceptible to experiencing secondary health impacts due to disasters. Owing to the lack of an established unified system for larger organisations, including the national government, to analyse and disclose such data, even in the case of large-scale disasters in Japan, the details of fatalities may not be available. As a result, obtaining detailed information on the circumstances of death, patients’ detailed medical records, degree of care provided, and details such as evacuation could be difficult. Additionally, due to lacking such information, comparing it with those of other disasters is challenging. For instance, considering the 2024 Noto peninsula earthquake, although 30 disaster-related deaths were reported, the causes of death were not disclosed. Despite ensuring careful handling of personal information, establishing a comprehensive database of disaster-related deaths remains an urgent task to minimise the impacts of future tragedies.

Finally, disaster-related deaths not only occur in the months immediately following a disaster but also can occur years after the event [7–9]. In regard to the case of the Fukushima Daiichi nuclear accident, research has reported disaster-related deaths due to repeated environmental changes several years after the incident [8]. According to data from Minamisoma, Fukushima Prefecture, which was affected by the Fukushima Daiichi nuclear accident, 41.0% of disaster-related deaths in Minamisoma occurred within three months after the disaster, 21.2% within three to six months, and 37.8% over six months. However, accessing data such as medical records of individuals who died several years after the disaster can be challenging due to issues with the retention period of medical records. Consequently, identifying cases of disaster-related deaths can be difficult; the number of such deaths may be underestimated. Furthermore, insufficient information sharing exists among experts regarding the long-term impacts of disaster-related deaths. Prior to the Fukushima Daiichi nuclear accident, the Nagaoka Criteria, which certified deaths within six months of a disaster as disaster-related deaths, had been used as the standard for recognising disaster-related deaths. However, this standard was updated in the wake of the Great East Japan earthquake and the Kumamoto earthquake. In 2024, the Minamisoma Criteria, which were used to officially recognise disaster-related deaths in the Great East Japan Earthquake and Fukushima Daiichi nuclear accident, were made public. However, these criteria are not yet recognised among experts and committee members involved in certifying disaster-related deaths. Consequently, there are cases wherein instances of disaster-related deaths occurring in the medium-to-long term after a disaster are not adequately recorded.

There are several important considerations when interpreting data on disaster-related deaths in Japan, as outlined above. Globally, the frequency of disasters continues to rise; with the ageing population, the indirect impacts of disaster-related deaths are expected to become significant [10]. Therefore, establishing unified criteria for the certification of disaster-related deaths based on medical evidence and lowering the barriers for bereaved families to apply for certification recognition of such deaths is necessary. Additionally, creating a data system for collecting detailed information on disaster-related deaths while considering the protection of personal information is also essential. By promptly initiating these measures, reducing the number of victims who perished in disaster-related deaths in future calamities might be possible.
